# The grant is dead, long live the data - migration as a pragmatic exit strategy for research data preservation

**DOI:** 10.12688/wellcomeopenres.15341.2

**Published:** 2019-09-23

**Authors:** Tomasz Zielinski, Johnny Hay, Andrew J. Millar

**Affiliations:** 1 SynthSys and School of Biological Sciences, University of Edinburgh, Edinburgh, EH9 3BF, UK; 2 EPCC, University of Edinburgh, Edinburgh, EH9 3FD, UK

**Keywords:** Data sharing, research data management, sustainable data infrastructure, exit strategy, research funding

## Abstract

Open research, data sharing and data re-use have become a priority for publicly- and charity-funded research. Efficient data management naturally requires computational resources that assist in data description, preservation and discovery. While it is possible to fund development of data management systems, currently it is more difficult to sustain data resources beyond the original grants. That puts the safety of the data at risk and undermines the very purpose of data gathering.

PlaSMo stands for ‘Plant Systems-biology Modelling’ and the PlaSMo model repository was envisioned by the plant systems biology community in 2005 with the initial funding lasting until 2010. We addressed the sustainability of the PlaSMo repository and assured preservation of these data by implementing an exit strategy. For our exit strategy we migrated data to an alternative, public repository with secured funding. We describe details of our decision process and aspects of the implementation. Our experience may serve as an example for other projects in a similar situation.

We share our reflections on the sustainability of biological data management and the future outcomes of its funding. We expect it to be a useful input for funding bodies.

## Introduction

Open research, data sharing and data re-use have become a priority for publicly- and charity-funded research, as expressed for example in the UK Concordat on Open Research ^1^. Data re-use depends on reliable metadata: a detailed description of the experimental conditions, materials used, handling procedures and analysis methods. Data management goes beyond the safe storage of data, because metadata acquisition and data discovery are equally important aspects for effective digital preservation ^2– 4^. This creates a need for computational resources that can deliver such features.

Funding bodies acknowledge that data management carries significant costs and allow budgeting for data stewardship. For larger projects this permits the development of systems suitable for a particular research domain, by supporting specific data models or streamlining metadata collection. This occasionally results in the formation of a small community resource, sometimes described as “boutique repository”. Unfortunately, while it is possible to fund data infrastructure for a project, currently, there are few schemes that could support a resource beyond the timeline of the initial grant ^5^. The common approach is to cover maintenance costs by “tunnelling” funds from related projects. That is not a sustainable model and puts at risk the very data that the original grant paid to preserve.

The increasing demand for data archiving induced the creation of general repositories (e.g. Figshare ^6^, Zenodo ^7^, Dryad ^8^) and also institutional repositories (e.g. University of Edinburgh DataShare ^9^, UK Data Archive ^10^). They may lack flexibility to support all the various needs of an active project, but they are valid alternatives for data preservation. We propose to address the sustainability problem and mitigate the risk to *boutique* data by implementing an exit strategy in the form of data migration to a larger, public repository with secured funding.

## Problem description

PlaSMo stands for ‘Plant Systems-biology Modelling’ and the PlaSMo portal (plasmo.ed.ac.uk) was envisioned by the plant systems biology community during a BBSRC and GARNet workshop in July 2005. The initial 2-year development was funded as part of BBSRC's Bioinformatics and Biological Resources call in 2008 and then supported by the European Commission's FP7 Collaborative Project TiMet (2010–2015).

The PlaSMo portal (henceforth referred to simply as ‘PlaSMo’) became a central resource for diverse plant models: general crop models, organ-level models or complex multi-component plant system models. At the time of its creation it was a unique resource for managing and sharing plant models, many of which were refactored into common, declarative languages (SBML or SimileXML). The PlaSMo repository contained over 100 described models and nearly 400 data and model files.

The main features of PlaSMo were:

- Support for multiple XML model formats: SimileXMLv3, SBML Level 2 versions 1–4, Cytoscape XGMMLv1, SBGN-MLv1
- Validation of the model format
- Managing multiple versions of the model
- Each version could have its own assets: definition file, supporting data, graphical representation, bibliography, description and comments
- Public, private or group access
- Free text search

The PlaSMo portal was implemented as a typical Java web application of its time: Apache Struts 2 as the Model-View-Controller (MVC) framework with Java Server Pages (JSPs) deployed on Apache Tomcat. The choice of Java as the language and technology stack proved to be robust and convenient. For example, the backend database was migrated from DB2 to MySQL, new model formats were added, and the Struts framework major version upgraded, all as ad-hoc tasks without the original developer present. Such tasks benefited from Java features such as strong typing, rich exception handling, well-defined JAR dependencies and IDE support.

Nevertheless, there were costs in providing such a public service including system administration, software development for occasional updates and user support. The Struts MVC framework had to be updated in a timely manner due to security concerns. There were critical vulnerabilities discovered in Struts that could permit arbitrary code execution and we observed attempts to exploit them just 8 hours after their disclosure. After the funded interval, all this work was performed as an in-kind contribution.

We noticed that PlaSMo had not been attracting new users. Its user interface was outdated, and the researchers had gained other facilities for sharing, like wikis or general repositories. It seemed that the value of the PlaSMo project was in its data rather than in its portal, hence, we decided to migrate the PlaSMo content into an alternative repository.

## Decision process

We planned to use a repository designed specifically for biological data instead of a general one like Figshare, Zenodo or University of Edinburgh DataShare. The general resources have no features relevant to biological data (e.g. model types, difference between model and data), they also tend to have a “flat” organization structure built around a concept of datasets. We wanted to preserve the “community” aspect of PlaSMo by having its resources grouped together and available for exploratory browsing. There is a dedicated repository for biological models: BioModels ^11, 12^ but it accepted only public (usually published) models and (at the time) was restricted to those in SBML format, whereas PlaSMo supported earlier stages of private model development and sharing among collaborators.

We chose FAIRDOMHub as the resource to host PlaSMo data ^13^. FAIRDOMHub is powered by the SEEK platform for managing systems biology data. SEEK (or FAIRDOM-SEEK) software was developed as part of the SysMO project, a 6-year trans-European initiative of over 100 biological research groups ^14^. We had previously evaluated SEEK from the perspective of handling plant models, so we knew that SEEK’s features aligned well with PlaSMo capabilities ^15^. SEEK organizes assets following the Investigation, Study, Assay (ISA) structure ^16^, offering user friendly navigation over the ISA tree. We could preserve the PlaSMo identity, utilizing the additional Project concept in SEEK. Below, we refer to FAIRDOMHub when we discuss the public web data repository and to SEEK when we discuss the underlying software platform and its concepts.

We represented PlaSMo records as SEEK entities in the following way:

- Each version of PlaSMo model is represented as a separate SEEK Modelling Assay
- PlaSMo model file becomes SEEK Model
- PlaSMo images and data files become SEEK DataFiles
- SEEK Model and DataFiles are linked to a corresponding Modelling Assay
- Metadata which is not easily represented in SEEK (e.g. comments) are appended to the description text of the Modelling Assay
- For each PlaSMo model a SEEK Study is created, and the Modelling Assays representing different versions of the model are linked to that Study
- For each user who deposited a model, a SEEK Investigation is created in their name, and all Studies representing their models are linked to that Investigation (see below)
- A SEEK project named “PlaSMo Model Repository” is created and all the Investigations, Studies, Assays, Models and DataFiles are linked to it
- All SEEK entities generated for public PlaSMo models are visible to anyone in SEEK
- For private PlaSMo models the descriptions of SEEK Studies and Assays are visible to anyone in SEEK but the actual content of Model and DataFiles is hidden

The main difficulty was how to handle permissions and ownership. SEEK has a very rich and flexible access control model (in our opinion, it is the best permission model we have seen so far) and SEEK assets can be linked to user profiles as their contribution. However, to benefit from these features we would need to have SEEK accounts for all the PlaSMo users.

We could not create matching FAIRDOMHub accounts for PlaSMo users: a) we were not entitled to perform such actions on behalf of the users, b) some users already had FAIRDOMHub accounts to which they would want their assets linked. To avoid contacting all the users with a request to create FAIRDOMHub accounts, we assumed a simplified approach.

Firstly, the creator of a PlaSMo model is recorded as a text label: “other contributors” in FAIRDOMHub. Secondly, for each PlaSMo user a SEEK Investigation is created with a title matching their name. The SEEK Studies representing PlaSMo models created by a user are linked to their Investigation. In that way the models of a particular PlaSMo user can be easily accessed by navigating to the SEEK Investigation named after them in FAIRDOMHub. It also solved the issue that SEEK requires a parent Investigation for all assets and we could not create a sensible convention for this based solely on PlaSMo model description.

If a person would like to claim their models, they would contact us with their FAIRDOMHub account and we would link the whole Investigation/Study/Assay tree to that account and grant the user the manager role for those assets. That way, the models’ creators could later manage their records using the SEEK UI.

PlaSMo users were always encouraged to link to their models using PlaSMo’s stable URLs. In order to preserve such links, we implemented a simple URL resolver that would redirect original PlaSMo references to the appropriate records in FAIRDOMHub.

**Figure 1.**
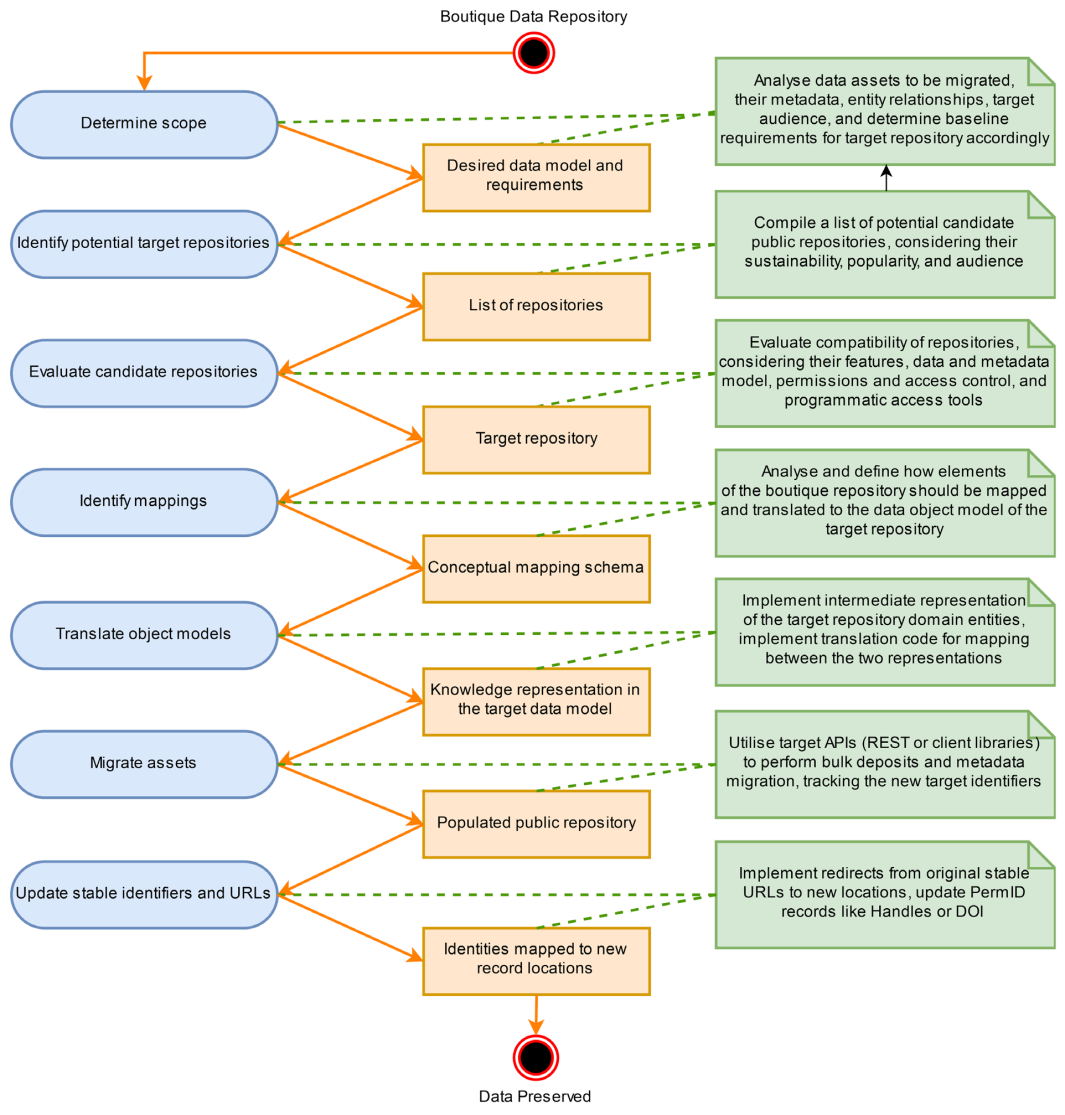
Implementation of an exit strategy for data preservation.

Figure 1 shows the generalized route for implementing an exit strategy for data preservation. 

## Implementation

We based the migration project on the existing code for the PlaSMo portal, in order to re-use the Data Access Objects (DAOs) and Data Object Model (DOM), so we only needed to implement the new data transfer logic.

We developed a Java client for programmatic communication with the SEEK REST API. Firstly, we used the available JSON request payload examples from SEEK REST API v1.7.0 ^17^ to generate a library of SEEK DOM JavaBean classes using the jsonschema2pojo v1.0.0 tool ^18^. We performed this step manually as it was a one-off project and we did not plan to keep the SEEK client in sync with the API in case it changes. Potential future work could make use of the jsonschema2pojo tool to regenerate these SEEK DOM classes automatically in the event of an update to the API.

The migration code iterates over PlaSMo models, extracting information required to generate JavaBeans corresponding to SEEK’s Investigations, Studies, Assays, Models and DataFiles entities. It then invokes the client methods to create the entities inside the SEEK instance, which serialize the JavaBean objects into JSON and submit them to the API via authenticated HTTP POST requests. During our initial tests, not all of the REST calls were consistently successful, for example sometimes we observed HTTP status 500 errors caused on the server by SQLite3::BusyException or AbstractController::DoubleRenderError. For that reason, we decided to record the API calls in a way that would allow them to be ‘replayed’ if needed without a risk of creating duplicate entities, always yielding a consistent ISA tree within a SEEK instance.

We used a local SQLite database to store the SEEK API calls, which was indexed with a GUID based on PlaSMo model UID and recorded the entire JSON payload and HTTP response for each entity in the ISA tree. This request logs database was also later used to create the mapping between PlaSMo URLs and FAIRDOMHub records (see below).

The FAIRDOMHub user interface currently does not allow for setting properties (e.g. permissions) on the whole ISA tree, a feature necessary for our migration strategy. We implemented such bulk operations in a separate Java project, which retrieves part of the ISA tree and sets the required properties – recursively through all child entities, if desired – using the Java API client.

The recorded API calls were used to generate a mapping between PlaSMo and FAIRDOMHub identifiers. The mapping was stored as a simple csv file for ease of potential future updates. This mapping is used by PlasmoMapper (a simple SpringBoot application) which resolves original PlaSMo URLs and redirects to the correct records in FAIRDOMHub.

## Results

We performed the migration on 10th of January 2019. All the information from the PlaSMo portal are available under the PlaSMo project on the FAIRDOMHub. The migration process was smooth and we did not experience any problem with the API calls. It seems that the SEEK instance on the FAIRDOMHub production server is very robust and it handles all the requests flawlessly, unlike the test SEEK’s Docker containers that we used during development.

**Figure 2.**
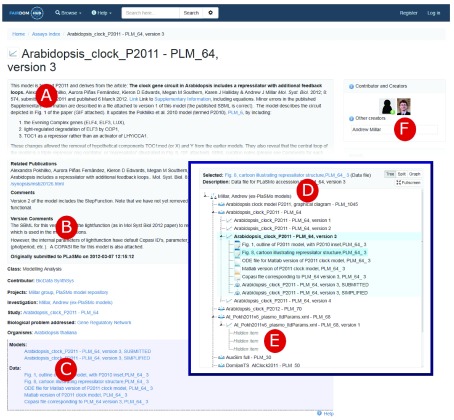
Screenshot (edited) presenting the FAIRDOMHub record for version 3 of PlaSMo model 64. **A**) Description of model 64 created from the main PlaSMo metadata; **B**) Version 3 specific details; **C**) List of linked model and data files; **D**) the navigation tree for models, versions and their data files; **E**) for private models the data and model files are hidden but the main metadata record is visible; **F**) link to the FAIRDOMHub user profile of the owner of the original PlaSMo model.

Figure 2 shows the FAIRDOMHub record for version 3 of PlaSMo model 64 (Arabidopsis_clock_P2011) represented as SEEK Assay 840. The description contains the merged version specific information with the details from the main PlaSMo record ( Figure 2 A, B). The other versions of that model are stored as the sibling Assays 838-841 ( Figure 2 C). Each version has the list of related model and data files ( Figure 2 C, D). All the model artefacts have been linked to the model owner profile in FAIRDOMHub ( Figure 2 F) by running the developed SEEK Bulk Update after the migration process.

All possible PlaSMo URLs are being redirected to the corresponding records in FAIRDOMHub, for example, the main link to the model 67: http://plasmo.ed.ac.uk/plasmo/models/model.shtml?accession=PLM_64 is redirected to Study 494; the version 3 of the model: http://plasmo.ed.ac.uk/plasmo/models/model.shtml?accession=PLM_64&version=3 to Assay 840 and the file containing Matlab version of this model: http://www.plasmo.ed.ac.uk/portal_data/data/PLM64/data/98mod_P2011.m to DataFile 2499.

We believe that the plant systems biology community will benefit from the PlaSMo models migration. The models are readily available for discovery by the larger userbase of FAIRDOMHub and models can be linked to experimental data. The potential for discovery is additionally enhanced by visibility of all the descriptions even of the private models, though for private models, the actual files are not accessible. That paves the way to potential collaborations without compromising the confidentiality of the data and is only possible due to SEEK’s rich permissions model. We note that this capability fulfills the stringent data sharing guidelines of UKRI-EPSRC.

We also feel that the migration boosted the profile of FAIRDOMHub as a community resource for data management and sharing. As shown in Table 1, transfer of the PlaSMo models substantially increased the number of available modelling assets (75% increase in model files). The effect of scale is an important aspect for attracting new users and the inclusion of plant models may popularise FAIRDOMHub among modellers.

**Table 1.** The total counts for each ISA entity type as they were in FAIRDOMHub before and after the PLaSMo models migration.

ISA Entity	Pre-Migration Total	Post-Migration Total	Total from PLaSMo	Percentage Increase
**Investigation**	197	212	15	7.6
**Study**	383	470	87	22.7
**Assay**	618	738	120	19.4
**Model**	255	446	191	75.0
**Data File**	1908	2097	189	9.9

## Discussion: Sustainability of Biological Data Management

We imported all the research assets from the boutique PlaSMo resource into a larger community resource: FAIRDOMHub. The migration process became feasible only after the developers of the underlying software, FAIRDOM-SEEK, released their write API in 2018. This illustrates the importance of write APIs for data management and system integration. Even with the API, the process involved laborious creation of code artefacts and mapping between concepts. We next consider how these might be avoided.

The enterprise world solved the issue of systems integration twenty years ago using SOAP ^19^ and WSDL ^20^ for web services. The enterprise ecosystem of JavaEE and .NET applications offered streamlined or even transparent generation of DOM, clients and endpoints based on the formal contract defined in XML documents. Unfortunately, the popular, new programming languages of the web, like Python, Ruby and JavaScript, lacked good support for XML and enterprise features, and as a result XML Web Services fell out of fashion. Additionally, the main driver behind the REST API is data consumption by a JavaScript UI, which has made JSON into the default exchange format. The lack of formality in the JSON REST API has been recently addressed by JSON Schema ^21^, OpenAPI ^22^ and JSON:API ^23^ tools, which should permit a similar level of automation as was achieved earlier by XML Web Services.

Semantic Web technologies and the Resource Description Framework (RDF) ^24^ specifically are meant to solve the issue of mapping between concepts. Indeed, SEEK represents its content as a knowledge graph in its backend, RDF triple store. However, the semantic mapping will only work automatically if both source and destination are using the same underlying ontologies. For example, SEEK uses the standard Dublin Core ^25^ for provenance terms but a custom JERM ontology ^26^ to document assets. Supervised translation and mapping between concepts or ontology terms seems unavoidable for the foreseeable future.

The problem of sustaining funding for research data resources is a general one, which has affected even the foundational resources of large communities such as The Arabidopsis Information Resource (TAIR) ^27^. Alternative funding models have been considered, including institutional and individual subscriptions, freemium, licencing for commercial use, advertising, crowdfunding or donations ^28– 34^. However, these are generally applicable only to services with a large user-base, not to specialised, boutique resources such as PlaSMo. The experience of shutting down such a community repository, while preserving its data, challenges some popular views of the feasibility of Research Data Management. For example, the successful migration of all the PlaSMo data to FAIRDOMHub could suggest that there is no need to fund any new systems for data management. A future project like PlaSMo should simply use the existing FAIRDOMHub from the start.

### Should funders still fund new software for data management?

In short, we believe the answer is yes, because this software can support and motivate both data sharing and research productivity.

Convincing researchers to invest the effort necessary to describe and deposit their data into a repository is the most difficult aspect of data management and a limiting factor in the wider adoption of data sharing. Data sharing can be achieved by using either “a stick” or “a carrot” approach.

The most successful “sticks” are strictly-enforced publication policies, as illustrated by the domain-specific requirements to deposit protein structural data (as in Protein Data Bank ^35, 36 ^), sequencing data (as in GenBank ^37, 38^) or transcriptomics data (as in ArrayExpress ^39, 40 ^). However, these repositories handle only narrow or single data types; there is consensus within each community on the minimal information criteria; in our opinion, these are the “easy” cases. For example, pdb files are in practice self-contained with metadata principally generated by equipment or processing software and require minimal interference from a scientist. Alternatively, the deposited file might represent all the results of a large, expensive experiment (e.g. a microarray study), so the effort in preparing and describing the data for deposit is small relative to the total effort in the experiment.

The “hard” cases include those that require detailed, user-generated information, for example a description of the biological materials and the experimental treatment of a specimen before measurements began. Hard cases will have multiple, complex experimental factors that vary significantly between samples, though each sample returns a modest data volume. Data consumption can also be harder in these cases, where data volumes and the complex relationship between individual entries complicate retrieval and analysis.

The current incentives (“carrots”) for data sharing are weak, considerably delayed in time and often accrue more to group leaders than to contracted researchers, hence they do not encourage widespread adoption of sharing practices ^41^. An alternative approach is to incorporate data management into the daily research workflow, by providing immediate value to data producers in the form of increased productivity, specialized processing, visualisation or data aggregation. For example, the BioDare repository is widely used within the circadian community, but researchers primarily use the resource to access specialist software tools to analyse and visualise their timeseries data, so the fact that BioDare datasets are simultaneously deposited in the public domain is in reality a side effect of the researcher’s core activity ^42, 43^. This level of tool customization and integration is project/domain specific ^44, 45^ and not possible with general repositories. Consequently, we expect such “carrots” to be rare among repositories that cater for many research domains, such as institutional repositories.

User friendliness is the most important characteristic for successful data management. The development of user friendly solutions that facilitate research (providing the specialist “carrots” we describe above) remains a valid case for new funding.

It is worth noting, that data management solutions may not need to be built entirely from scratch. One could leverage features of existing products (like for example SEEK or OpenBIS ^46^) and create plugins or integrate with them. Which approach is most cost effective and productive must be evaluated case by case, depending on the available know-how and expected user experience.

A positive example of promoting data management is the Wellcome Trust “Research Enrichment – Open Research” scheme ^47^, which funds small, add-on projects for existing grant holders to enable open research and data sharing. By presenting this as add-on funding, the implementation of data sharing is perceived as an additional opportunity, rather than in competition with core scientific activities for funding.

### Can research data repositories be self-sustaining?

In majority of the cases, no.

The idea that domain-specific resources could often be maintained from subscription fees is unrealistic ^29, 33, 34^:

1. There is a problem of scale. If we advocate for resources that address particular needs of scientific projects, the underlying user base or even the entire research community may be too small to sustain a public repository financially. Conversely, repositories catering to a diverse community may gain sustainability but lack user uptake.
2. Data producers already commit their time and make a substantial effort to prepare data for deposit, so we cannot expect them to be charged for deposit on top of the work they do to contribute their data.
3. Charging for access to data is against the spirit of open research and data re-use. Funding agencies generally require the public release of results, or are moving to do so, and such a model would be an infringement of their policies.
4. Micropayment models, with small fees for extra features that one might use (e.g. a minted DOI or a longer embargo period) could be acceptable to the users but it is impractical in the academic world. Research groups often do not have credit cards to perform small payments automatically, but invoicing and accounting for such operations would be problematic and not cost-effective.

While it is possible to secure funding for a new project, there are currently few funding schemes to maintain existing data resources. Incremental improvements to existing resources can also be problematic, as they may not offer the novelty and impact to compete with new infrastructure. However, maintaining existing resources may be as important as funding new science as it is the only way to enable data re-use. At the same time, the data repositories should gather metrics in order to demonstrate their value, for example numbers of active users, visits and downloads.

### How to deliver data longevity?

Our PlaSMo migration demonstrates that data longevity can be achieved by implementing an effective exit strategy. In our case, we found a close match for our metadata model in the FAIRDOMHub. If a sufficient match is not available, it is always possible to find a generic destination that can at least archive all the data. The implementation of a migration involves additional costs but in the long term, it is usually cheaper than maintaining a running resource.

The biggest value of data repositories lies in their data; hence, we would recommend creation of funding opportunities that could be used to “rescue the data”. Data migration could constitute part of the income agreement for maintaining destination repositories. For example, a repository could receive extended funding on the condition that it would implement the adoption of data from other projects.

Currently, data migration seems to be an inevitable reality of data preservation. Permanent identifiers (like DOIs or handles) which can resolve to the actual location facilitate this process. If PlaSMo models had DOIs we would not need to deploy PlasmoMapper to handle the original URLs. Unfortunately, participation in permanent identifier schemes can incur additional financial costs, which paradoxically may accelerate the demise of a repository.

In Horizon2020, the EU funded various initiatives to provide European Research e-Infrastructure, and participating consortiums offer permanent identifiers as part of their services (EUDAT ^48^, ePIC ^49^). Sadly, although the initiative is already centrally funded, the identifiers (handles in this case) were provided only as a paying service. More domain-specific projects have been funded to offer free IDs, such as the identifiers.org service at EMBL EBI, which is now linked to the ELIXIR project. We believe that permanent identifiers should be available free of charge not only for data projects but even for individuals as a public service, similar to street address systems.

## Conclusions

We shared our experience in securing the PlaSMo project’s legacy and assuring data longevity by successfully implementing an exit strategy in the form of data migration. We believe that further progress in open research and data sharing can only be achieved by integration between different resources that together can be incorporated into research workflows. We are concerned over the existing funding opportunities for data management and how they might put at risk the safety of scientific data.

## Reuse potential

The Java Client for the SEEK REST API and the bulk property setter, described here, can be of value for other projects. The client can be used to integrate other Java projects with SEEK, for example to automate data deposition. The bulk property setter compensates for a currently missing feature in the SEEK UI. Running the setter is currently the easiest way to publish multiple datasets constituting a research outcome. For these reasons we made the relevant code available as two separate packages.

## Data availability

### Underlying data

All data underlying the results are available as part of the article and no additional source data are required.

## Software availability

**Java Client for SEEK API**

Source code is available from: https://github.com/SynthSys/Seek-Java-RESTClient

Archived source code at time of publication: https://doi.org/10.5281/zenodo.3250951 ^50^

Licence: MIT

**SEEK Bulk Update**

Source code is available from: https://github.com/SynthSys/Seek-Bulk-Update

Archived source code at time of publication: https://doi.org/10.5281/zenodo.3250959 ^51^

Licence: MIT

**PlaSMo portal**

Source code is available from: https://github.com/SynthSys/PlasmoPortal

Archived source code at time of publication: https://doi.org/10.5281/zenodo.3250855 ^52^

Licence: MIT
