# Supplementary material for: The grant is dead, long live the data - migration as a pragmatic exit strategy for research data preservation
Source: Wellcome Open Res. 2019 Sep 23;4:104. Originally published 2019 Jul 2. [Version 2] doi: 10.12688/wellcomeopenres.15341.2 (PMC6652102; doi:10.12688/wellcomeopenres.15341.2)
Supplement: Peer review history

# Peer review: Reviewer response for version 1

**DOI:** 10.21956/wellcomeopenres.16751.r35895

**Published:** 2019-07-22

**Authors:** Robert Davey

**Affiliations:** 1 Earlham Institute, Norwich, UK

The authors describe a mechanism for preparing and executing a "handover" strategy to ensure long(er) term hosting and sustainability of biological datasets. This is presented through the authors' experiences in carrying this out for their own project, PlaSMo. Whilst there isn't much that is particularly novel about this article, it could be incredibly useful for those groups or consortia that are approaching a similar cliff face in funding support for their data repository or infrastructure.

 My main concern is that the article doesn't really cover the breadth of information in the literature concerning other projects that have gone through a similar procedure. Araport and TAIR is one such example, and relevant to the funding sustainability argument. The references provided are generally suitable, but many of them (those that aren't referencing a public data repository) are from one of the authors. I understand this from the point of the use case - it's sensible to provide supporting evidence for their tools. However, there are other recent publications regarding plant-based information resources that could be mentioned to cover a wider scope. A more comprehensive view of issues in other plant science circles, maybe even other domains of science, would be good, given the article's quite broad title. There is a huge amount of literature on open science, reproducibility, the availability of resources and how to handle data openly to ensure longevity.

 That said, the article makes salient points that are worthy of thought. This article would be of use to funders currently, who are facing a real problem on their hands as data infrastructure is increasingly needed at a time when funds for creating and sustaining these resources aren't increasing accordingly.

 The article is clearly written and very easy to follow with concise points and no lengthy prose. It was nice to read, so the authors should be commended here.

 I would have liked to have seen a critical view of their own exit strategy. Key parts of their strategy are often overlooked in practice when considering day-to-day actions of scientists and infrastructure developers. Are there pros/cons to this strategy, and are there other solutions available that can assist with certain "pain points"? The mapping/translation/knowledge representation process could be expensive and time-consuming. Are there tools that can assist? If not, why not?

 This is particularly relevant when discussing the carrots and sticks argument. What are the "hard cases"? High-throughput plant phenotyping could be one such example, as even though there is developing consensus, there are still many issues around the longevity and usefulness of storing these datatypes. Exploring this a little more may give a helpful counterpoint to why the easy cases aren't simply about support for narrow/single datatypes. Handling this heterogeneity of data outputs, automated or manually curated, is a key issue for many scientists, and indeed feeds into the user friendliness issue.

 Is the ubiquity of paid-only permanent identifiers true for all EU projects? I wasn't aware of this, so a reference to this would be useful for readers to understand this further. Some data repositories can provide DOIs for free, so is there a part of the exit strategy that could include wholesale deposition/mirroring of datasets within such a repository?

I confirm that I have read this submission and believe that I have an appropriate level of expertise to confirm that it is of an acceptable scientific standard.

# Response

**Published:** 2019-09-17

**Authors:** Tomasz Zielinski

**Affiliations:** University of Edinburgh, UK

Thank you for your extensive and constructive review. Our detailed responses are listed below, with pointers to the revised paper text.

**Reviewer: **“My main concern is that the article doesn't really cover the breadth of information in the literature concerning other projects that have gone through a similar procedure. Araport and TAIR is one such example, and relevant to the funding sustainability argument. The references provided are generally suitable, but many of them (those that aren't referencing a public data repository) are from one of the authors. I understand this from the point of the use case - it's sensible to provide supporting evidence for their tools. However, there are other recent publications regarding plant-based information resources that could be mentioned to cover a wider scope. A more comprehensive view of issues in other plant science circles, maybe even other domains of science, would be good, given the article's quite broad title. There is a huge amount of literature on open science, reproducibility, the availability of resources and how to handle data openly to ensure longevity.”

 To address these comments, we have added new text at the start of the Discussion. One paragraph (starting “ *The problem of sustaining funding for research data resources is a general one,…*") describes the funding alternatives, with some additional references including the example of TAIR, which we agree is a relevant example.

 Note, first, that this article was accepted in the Research Note format, described as a “one figure paper” in the Wellcome guidelines. It was not possible or appropriate to cover the whole landscape of open research and repository funding. Rather, we presented the particular challenges that we faced as a case study for a broadly-relevant problem, how we addressed these issues and what reflections this case provoked on the sustainability of research data sharing more generally. We ensured that the methods and conclusions are as general and reusable as widely as possible. The title reflects our use case and the content of the paper.

 Second, we focus on boutique repositories that address specific needs of a particular community, in this case crop and plant systems modelling. This is a far smaller resource than Araport and TAIR, which serve all of plant science and some of the broader genomics community. Their much larger scale made a subscription model possible for TAIR, which would not work for smaller but still valuable resources, as we comment later in the discussion.     

**Reviewer: **“The references provided are generally suitable, but many of them (those that aren't referencing a public data repository) are from one of the authors.”

 We had cited only two of our articles among a total of 31 references, plus 3 links to code snapshots that were requested by the Wellcome editors. This did not seem excessive.

**Reviewer: **“I would have liked to have seen a critical view of their own exit strategy. Key parts of their strategy are often overlooked in practice when considering day-to-day actions of scientists and infrastructure developers. Are there pros/cons to this strategy, and are there other solutions available that can assist with certain "pain points"? The mapping/translation/knowledge representation process could be expensive and time-consuming. Are there tools that can assist? If not, why not?”

 We largely agree that parts can be overlooked, which is why we felt it worthwhile to document the process and the reasoning in detail, and we focussed on a specific case to do so. The new Discussion text addresses tools to ease the pain points (starting “We next consider how these might be avoided“), specifically automating the code generation and knowledge mapping, with a comparison of current and earlier tools. We had discussed alternatives for particular stages but for the process overall, the alternatives are not very helpful. They amount to either providing the service without financial support (the *status quo ante*) or closing the service and with it the data access.

**Reviewer: **“This is particularly relevant when discussing the carrots and sticks argument. What are the "hard cases"? High-throughput plant phenotyping could be one such example, as even though there is developing consensus, there are still many issues around the longevity and usefulness of storing these datatypes. Exploring this a little more may give a helpful counterpoint to why the easy cases aren't simply about support for narrow/single datatypes.”

 We now discuss the “hard” cases in a paragraph starting “The “hard” cases include those that require detailed, user-generated information...”. Phenotyping data are “big data” both in volume and velocity, so the main challenge is the infrastructure (e.g. storage and bandwidth). Data deposition is relatively easy as data are collected automatically and there is minimal need for human curation. Data reuse will depend on developing a consensus on the metadata for recorded values, and as the reviewer notes this consensus is emerging but not complete. We see the main challenge in this kind of data in reassembling suitable datasets for analysis from a large volume of related recordings.

**Reviewer: **“Is the ubiquity of paid-only permanent identifiers true for all EU projects? I wasn't aware of this, so a reference to this would be useful for readers to understand this further.

 As we state in the manuscript, permanent IDs are not freely available in general. We added examples of partially funded EU projects that provide PID as a paid service. The costings in detail vary considerably, as the DOI FAQ implies: “The cost of registering new DOI names depends on the services you purchase”. Handle system has a different charging structure, see http://www.handle.net/payment.html. More domain-specific projects have been funded to offer free IDs, such as the identifiers.org service at EMBL EBI, as we note in the Discussion.

**Reviewer: **“Some data repositories can provide DOIs for free, so is there a part of the exit strategy that could include wholesale deposition/mirroring of datasets within such a repository?”

 Having a new, free DOI minted by a destination repository does not help in preserving the previous links or ID, whereas having a PID to start with allows a transparent redirect to the new location of a migrated resource.

# Peer review: Reviewer response for version 1

**DOI:** 10.21956/wellcomeopenres.16751.r35896

**Published:** 2019-07-16

**Authors:** Helen Ougham

**Affiliations:** 1 Aberystwyth University, Aberystwyth, UK

This paper represents both a useful case study in migrating a biological research resource - in this case, the plant models repository PlaSMo - to a new public repository - and a description of the serious practical, especially financial difficulties, of maintaining specialised datasets. Data sharing, though both inherently desirable and a requirement of many funders, is not a trivial exercise in cases where no major international repository/database is available; there is often a real danger that research outputs will be either lost or made available in a form unsuitable for effective re-use.

 The original PlaSMo portal, which used computational features appropriate to its time, was designed as a resource for plant and agricultural researchers to access a range of plant models, some very new, but others long-established but in danger of being lost as their originators retired and, in some cases, source code became difficult to access. It was always intended that those in the rapidly-developing systems biology community should be able to capitalise on these models to assist them in extending modelling of biological processes from the cellular to the organ, whole plant and ultimately crop level. Now, some years later, the number of plant systems biology models is much greater, and it made sense for the authors to migrate the models and datasets originally available through PlaSMo to a contemporary repository already accommodating many systems biology models and in use by current systems biologists. At the same time, this would secure the classic crop models for the plant physiologists and breeder. Carrying out this migration addressed issues of potential security threats as well as reducing the overhead inherent in maintaining the PlaSMo resource in its original form.

 The paper clearly sets out the rationale for the work, the steps taken, the tools used, and the form in which the original PlaSMo models and associated files are now to be found in FAIRDOM hub; the latter has grown considerably as a result, particularly with respect to the number of models available. Although certain aspects of the original PlaSMo have been lost (ability to run web-based simulations, for example), this is an inevitable consequence of the move and is unlikely to adversely affect most current users of the models.

 The paper is generally very well written; there are a few sections where the English reads a little as though it was written by a non-native speaker, but the meaning is always very clear.

 A couple of minor typos: there is one instance where PlaSMo is written PlaSMO, and this should be amended for consistency; and 'GeneBank' should be 'GenBank'.

 I did notice a spelling mistake on https://github.com/SynthSys/PlasmoPortal, where FAIRDOMhub is shown as FaridoHub!

 All the URLs in the article worked correctly at the time of this review.

 The table and the two figures are useful and appropriate. In Figure 1 the boxes on the right (in green) have 'folded over' corners which in some cases slightly obscure the text; this should be easy to address.

I confirm that I have read this submission and believe that I have an appropriate level of expertise to confirm that it is of an acceptable scientific standard.

# Response

**Published:** 2019-09-17

**Authors:** Tomasz Zielinski

**Affiliations:** University of Edinburgh, UK

Thank you for your very positive review. We have corrected the typos and the layout of Figure1 as suggested.
